# How does the macroenvironment influence brain and behaviour—a review of current status and future perspectives

**DOI:** 10.1038/s41380-024-02557-x

**Published:** 2024-04-24

**Authors:** Elli Polemiti, Sören Hese, Kerstin Schepanski, Jiacan Yuan, environMENTAL consortium, Elli Polemiti, Sören Hese, Kerstin Schepanski, Jiacan Yuan, Gunter Schumann, Gunter Schumann

**Affiliations:** 1 Centre of Population Neuroscience and Stratified Medicine (PONS), Department of Psychiatry and Clinical Neuroscience CCM, Charité-Universitätsmedizin Berlin, Berlin, Germany; 2 Institute of Geography, Friedrich Schiller University Jena, Jena, Germany; 3 Institute of Meteorology, Free University Berlin, Berlin, Germany; 4 Department of Atmospheric and Oceanic Sciences & Institute of Atmospheric Sciences & CMA-FDU Joint Laboratory of Marine Meteorology & IRDR-ICOE on Risk Interconnectivity and Governance on Weather/Climate Extremes Impact and Public Health, Fudan University, Shanghai, China; 5 Centre for Population Neuroscience and Stratified Medicine (PONS), Institute for Science and Technology of Brain-inspired Intelligence (ISTBI), Fudan University, Shanghai, China

**Keywords:** Psychiatric disorders, Neuroscience, Psychology

## Abstract

The environment influences brain and mental health, both detrimentally and beneficially. Existing research has emphasised the individual psychosocial ‘microenvironment’. Less attention has been paid to ‘macroenvironmental’ challenges, including climate change, pollution, urbanicity, and socioeconomic disparity. Notably, the implications of climate and pollution on brain and mental health have only recently gained prominence. With the advent of large-scale big-data cohorts and an increasingly dense mapping of macroenvironmental parameters, we are now in a position to characterise the relation between macroenvironment, brain, and behaviour across different geographic and cultural locations globally. This review synthesises findings from recent epidemiological and neuroimaging studies, aiming to provide a comprehensive overview of the existing evidence between the macroenvironment and the structure and functions of the brain, with a particular emphasis on its implications for mental illness. We discuss putative underlying mechanisms and address the most common exposures of the macroenvironment. Finally, we identify critical areas for future research to enhance our understanding of the aetiology of mental illness and to inform effective interventions for healthier environments and mental health promotion.

## Introduction

The environment refers to the broader ecological context in which an individual exists, interacts, and adapts [1], and may have direct and indirect effects on mental health [2]. It can be broadly divided into the ‘macroenvironment’, encompassing environmental characteristics at the neighbourhood or larger level, and the ‘microenvironment’, which relates to the individual psychosocial level [3]. The macroenvironment includes factors such as urbanisation, climate patterns, geological features, and ecosystem interactions, as well as socioeconomic disparity—all of which are undergoing rapid and dynamic changes. Urbanisation continues at unprecedented rates, with more than 50% of the population residing in cities [4], involving the expansion of infrastructure and shifts in land use patterns and population density. These alterations contribute to increased environmental pollution and decreased availability of natural spaces [4]. Climate change results in rising temperatures changed weather patterns, and extreme weather events [5]. These factors are interconnected, and changes in one may trigger or amplify changes in another.

Mental disorders ranked among the three leading causes of health loss globally and consistently contribute to over 14% of age-standardised years lived with disability during the past three decades [6]. It has been suggested that adverse macroenvironmental factors contribute to an increased risk of mental health disorders [7–9] and may account for more than 20% of population attributable risk of mental disorders [10, 11]. While extensive research has explored the influence of the microenvironment on brain and mental health, there has been growing awareness of the significance of the macroenvironment in recent years with emerging insights and findings that warrant further exploration. Mental illness may result from accumulated exposure to single or multiple environmental factors throughout the individual’s life course. In almost all cases, there is a complex interplay between risk and protective factors of micro- and macroenvironment.

In view of these complex dynamics, it is essential to understand how the macroenvironment contributes to the occurrence of mental illness and which are the neurobiological underpinnings of this relationship. In the following sections, we document the association of the macroenvironment with brain structure and function and attempt to connect these findings to potential risks of mental illness. We address the most common macroenvironmental exposures that encompass immediate environmental factors, such as air, noise and light pollution, proximal factors comprising regional socioeconomic characteristics, and distal factors, like urbanisation, natural spaces, and climate. These macroenvironmental exposures are mostly modifiable, presenting opportunities for interventions and strategies to promote the structural and functional integrity of the brain and mitigate the burden of mental illness.

## Search strategy and study selection

We conducted a literature search on the association between modalities of the macroenvironment and magnetic resonance imaging (MRI)-assessed brain structure and function in PubMed from January 1, 2010, to April 19, 2023. While different neuroimaging techniques offer unique advantages and insights, we focused on MRI studies due to our expertise in this area. We used predefined search terms (Supplementary Information), with no restrictions applied except for the filter [Humans]. In short, MeSH (Medical Subject Headings) terms and title/abstract text words related to environmental exposures were employed, including urbanisation, air, noise and light pollution, green space, blue space, regional socioeconomic factors, climate, weather extremes, combined with MRI-detected brain changes in structure and function. The reference lists of relevant systematic reviews identified in our formal search were hand-searched for relevant literature. Furthermore, studies known to the authors were added.

The present review is not exhaustive in covering all potential macroenvironmental factors influencing brain and mental health. Exclusion criteria were applied to focus on macroenvironmental factors that are pervasive and modifiable at the general population level. Publications on animal models and cell lines were excluded. Studies investigating the association between indoor air pollution, occupational hazards and neurotoxins with brain plasticity were further excluded due to their confinement in specific indoor environments, work-related settings, or lifestyle contexts. Finally, studies on natural disasters were excluded due to their sporadic occurrence and localised impact.

## Air pollution

Air pollution arises from natural phenomena, like dust storms or wildfires, and from human activities, such as industrial processes and transportation. It includes solid particles and liquid droplets suspended in the air, referred to as particulate matter (PM), and gases, like ground-level ozone, sulphur dioxide, nitrogen oxides (NO_x_: NO + NO_2_), carbon monoxide, polycyclic aromatic hydrocarbons (PAH) and others [12]. Each of these pollutants may have independent and potentially synergistic effects; however, the impact of exposure to a combination of air pollutants on human health is not well understood [13].

Air pollutants enter the body through the respiratory system, initiating a cascade of physiological and biochemical responses affecting different tissues and organs, including the brain [14, 15]. Pollutants can translocate across the blood-brain barrier, a critical protective barrier of the brain, potentially inducing systemic inflammation [14] and compromising the permeability of the blood-brain barrier itself [16]. Air pollution-related neuroinflammation has been associated with neurotoxicity, oxidative stress, and impaired control of inflammatory processes [17, 18]. The developing brain is particularly vulnerable to toxicants during two critical developmental periods, the foetal and early life, due to the limited barrier function of the placenta and blood-brain barrier, and potential toxicant transfer during breastfeeding [19]. This heightened vulnerability stems from the interference with fundamental neurodevelopmental processes, including neuronal growth, formation of synaptic networks, neuronal migration, and development of receptor numbers. These processes are most active during foetal and childhood stages, and disruptions due to pollutant exposure may alter brain development [20].

Prenatal and early childhood exposure to several components of traffic-related air pollution (TRAP), such as PM, PAH, airborne copper and organic carbon, appeared to influence brain development in later childhood and adolescence [21–29], including the corpus callosum [22, 29], limbic system [22, 27], nucleus accumbens (NAc) and cerebellum [22, 28] (see Table 1 for a detailed description). In addition to these structural changes, TRAP exposure has been associated with functional connectivity (FC) changes. Cross-sectional analyses among children and preadolescents primarily reported these changes in frontocortical areas and the default mode network (DMN) [30, 31].

**Table 1:** Studies on air pollution and MRI-detected alterations in brain structure and function.

Exposure period (Year)	Exposure duration (Year)	Period at MRI assessment (Year)	Pollutant/ Method of assessment	Brain alterations	Reference
Lifetime	Lifetime	Childhood Mean age ± SD: ~10.7 ±2.3 years 2 MRI assessments 1 year apart. Mean age ±SD at baseline: ~7.0 ±0.6 years	Lived in a highly polluted city vs low polluted city	Prefrontal WM hypersensitivity ↓ WM volumes in temporal and parietal (R) lobe at 1 year follow-up among children residing in a highly polluted city compared to children from a low polluted city	[250, 251] Mexico City and Polotitlán area study n ≈ 30
Childhood 9–10 years (2016–2018)	1 year (2016)	Childhood 9–10 years (2016–2018)	PM2.5 Spatiotemporal model	↓ SA in frontal pole (R), cuneus (L) ↑ SA in lateral orbitofrontal (R) ↓ CT in lateral orbitofrontal (L), superior frontal (L), inferior temporal (R), parahippocampus (R), rostral anterior cingulate (L), caudal anterior cingulate (L), posterior cingulate (L), isthmus (L), insula (R) ↑ CT lateral orbitofrontal (R), paracentral (R), middle temporal (L), rostral anterior cingulate (R), caudal anterior cingulate (R), posterior cingulate (R) ↑ volumes in accumbens (L), pallidum (R), thalamus (R) ↓ volumes in pallidum (L), putamen (L)	[33] ABCD study n = 10,341
Childhood 9–10 years (2016–2018)	1 year (2016)	Childhood 9–10 years (2016–2018)	PM2.5 Spatiotemporal model	↑ rN0 (nonlinear) in the cingulum hippocampal portion (L), uncinate fasciculus (L), and fornix (L) ↑ rN0 (linear) in the uncinate fasciculus (R), the fornix (R), superior longitudinal fasciculus (L) ↓ MD (nonlinear) in the anterior thalamic radiations (L), cingulum hippocampal portion (L), fornix (L), superior longitudinal fasciculus (L), uncinate (L), inferior longitudinal fasciculus (R), and uncinate (R) ↓ MD (linear) in the inferior fronto-occipital (L), inferior longitudinal fasciculus (L), cingulum hippocampal portion (R), fornix (R)	[32] ABCD study n = 7602
Childhood/Preadolescence 9–13 years	2 years prior to first MRI assessment	Preadolescence/ adolescence 11–15 years 2 MRI assessments 2 years apart	PM2.5 Spatiotemporal models	At 2 years follow-up higher PM2.5 exposure was associated with the following changes: ↑ WM volume in caudate/corpus callosum (L), cingulum (L), inferior fronto-occipital fasciculus, inferior frontal gyrus (R), inferior temporal gyrus (R) ↑ GM volume in precentral gyrus (L), cerebellum (L), medial orbitofrontal cortex ↓ WM volume in inferior temporal gyrus (L), angular gyrus (L), posterior thalamic radiation (L), middle frontal gyrus (L), hippocampal cingulum (L), postcentral gyrus (R) ↓ GM volume in insula (L), cingulate gyrus (R), caudate (R), cerebellum (L), fusiform gyrus, precentral gyrus, middle frontal gyrus	[34] San Francisco and San Jose Bay Area study n = 115
Prenatal	Whole pregnancy (PM2.5) 48-h during last trimester (PAH)	Childhood/Adolescence 6–14 years	PM2.5, PAH Spatiotemporal models (PM2.5) Personal air monitors (PAH)	**Exposure to PM2.5** ↓ WM surface in lateral pre/procentral gyrus, superior frontal gyrus, middle frontal gyrus (L), middle temporal gyrus (L), inferior parietal lobule (L), anterior cingulate cortex, posterior cingulate cortex (R) ↑ WM surface in medial and dorsal pre/procentral gyrus, medial superior frontal gyrus, lateral superior temporal gyrus (R), dorsal superior parietal gyrus ↓ CT in superior parietal gyrus, pre/procentral gyrus ↑ CT in superior frontal gyrus, inferior frontal gyrus (L), superior temporal gyrus (L), inferior temporal gyrus, middle temporal gyrus, inferior parietal lobule (L), anterior cingulate cortex, posterior cingulate cortex (R), fusiform and lingual gyrus ↑ FA in caudate, lenticular nucleus, insula, brainstem, thalamus, cingulate gyrus, superior corona radiate ↑ ADC in inferior fronto-occipital fasciculus, anterior corona radiata, vertical occipital fasciculus **Exposure to PAH** ↓ WM surface in inferior temporal gyrus, middle temporal gyrus, inferior parietal lobule (L), anterior cingulate cortex (R), posterior cingulate cortex (L) ↑ WM surface in pre/procentral gyrus, superior frontal gyrus, dorsal middle frontal gyrus, ventral fusiform gyrus, ventral lingual gyrus ↓ CT in superior frontal gyrus, middle frontal gyrus, inferior frontal gyrus (L), pre/procentral gyrus, superior temporal gyrus (R), middle temporal gyrus (R) ↑ CT in middle temporal gyrus (L), anterior cingulate cortex (R), fusiform and lingual gyrus ↑ FA in middle orbitofrontal gyrus, cerebellum, hippocampus, globus pallidus, putamen, thalamus, corpus callosum, internal capsule ↓ ADC in internal capsule, corpus callosum	[27] CCCEH study n = 332
Prenatal	1st, 2nd, 3rd trimester and whole pregnancy	Childhood/ Preadolescence 8–12 years (2012–2014)	PM2.5 LUR models Measurements from monitors at site were collected between Oct 2008 and Apr 2011	↓ volume in total, anterior and body corpus callosum with higher PM2.5 exposure during the 3rd trimester. Associations did not survive false discovery rate correction. No association for WM, GM and lateral ventricles	[29] BREATH n = 186
Childhood/Preadolescence (2012) 7–11 years 8–12 years	1 year (2012)	Childhood/ Preadolescence 7–11 years 8–12 years (2012–2014)	NO2, PAH, BPA, EC, copper Monitors at site Two 1-week periods separated by two semesters	**Exposure to PAH, BPA and NO2** ↓ Caudate volume No association for putamen and globus pallidus volumes **Exposure to EC** No association for caudate, putamen, and globus pallidus volumes **Exposure to copper** ↑ GM concentration in the caudate nucleus No association for putamen and globus pallidus ↑ FA predominantly in caudate nucleus ↓ rsFC between the frontal lobe opercula and the caudate nuclei, and vice versa	[30, 35, 36] BREATH study n ≈ 200
Childhood/ Preadolescence (2012) 8–12 years	1 year (2012)	Childhood/ Preadolescence 8–12 years	Pollution index: weighted average of pooled indoor and outdoor NO2 and EC Monitors at site Two 1-week periods separated by two semesters	↓ rsFC between regions belonging to the DMN ↑rsFC between the medial frontal cortex and the frontal operculum at the lateral boundary of the DMN ↓ deactivation in the supplementary motor area and somatosensory cortex in the study deactivation map	[31] BREATH study n = 263
Prenatal (2001–2006)	Whole pregnancy	Childhood 6–10 years	NO2, PMcoarse, PM2.5, PM2.5abs LUR models 2-week measurements in three different seasons (warm, cold, and intermediate) from monitors at site between Feb 2009 and Feb 2010	↓ CT in praecuneus (R), pars opercularis (R), pars orbitalis (R), rostral middle frontal (R), superior frontal (R), cuneus (L) with higher exposure to PM2.5 ↓ CT in lateral orbitofrontal (R) with higher exposure to PM2.5coarse ↓ CT in fusiform (L) with higher exposure to PM2.5abs	[22] Generation R study n = 783
Prenatal (2001–2006)	Whole pregnancy	Preadolescence 9–12 years	NOx, NO2, PM10, PMcoarse, PM2.5, PM2.5abs, PAH, OC, copper, iron, silicon, zinc, OP LUR models 2-week measurements in three different seasons (warm, cold, and intermediate) from monitors at site between Feb 2009 and Feb 2010	↑ volumes of putamen and pallidum with higher exposure to PMcoarse ↑ volume of cerebellum with higher exposure to PM10, PMcoarse, PM2.5, PM2.5ab ↓ volume of hippocampus with higher exposure to PAH, copper ↓ volume of amygdala with higher exposure to OC, silicon ↓ volume of corpus callosum with higher exposure to OP ↓ CT in postcentral gyrus (R) with higher exposure to OC (marginally nonsignificant) ↓ CT in rostral middle frontal gyrus (R) with higher exposure to copper and PM2.5abs (marginally nonsignificant) ↓ FA in forceps minor, corticospinal tract, superior longitudinal fasciculus (R) with higher exposure to PM2.5 ↑ MD in cingulum bundle, forceps minor, superior longitudinal fasciculus (L), inferior longitudinal fasciculus (L) with higher exposure to silicon ↑ rsFC between brain regions of the same brain hemisphere, predominantly in the auditory association, dorsolateral prefrontal, somatosensory and motor, anterior cingulate and medial prefrontal, dorsal stream visual, and insular and frontal opercular cortices with higher exposure to NO2	[22] Generation R study n = 3133
Childhood 6–10 years	1 year	Preadolescence 9–12 years	NOx, NO2, PM10, PMcoarse, PM2.5, PM2.5abs, PAH, OC, copper, iron, silicon, zinc, OP LUR models 2-week measurements in three different seasons (warm, cold, and intermediate) from monitors at site between Feb 2009 and Feb 2010	↓ volume of hippocampus with higher exposure to PMcoarse, OP ↑ volume of nucleus accumbens with higher exposure to zinc ↓ volume of corpus callosum with higher exposure to OC ↓ CT in lingual gyrus (L) with higher exposure to copper and OP (marginally nonsignificant) ↑ SA in precentral gyrus (R) with higher exposure to zinc and OP ↑SA in pericalcarine cortex (L) and precuneus (L) with higher exposure to zinc ↓ SA in pars triangularis (R) with higher exposure to PMcoarse (marginally nonsignificant) ↓ FA in corticospinal tract (L), uncinated fasciculus, superior longitudinal fasciculus (R), inferior longitudinal fasciculus (R) with higher exposure to NOx ↑ MD in cingulum bundle (L) with higher exposure to OP ↑ MD in cingulum bundle, forceps minor, superior longitudinal fasciculus, inferior longitudinal fasciculus, uncinated fasciculus with higher exposure to zinc	[22] Generation R study n ≈ 3000
Prenatal and childhood	Whole pregnancy and childhood at periods: 0–2 years 2–5 years 5–9 years	Preadolescence 9–12 years	NOx, NO2, PM10, PMcoarse, PM2.5, PM2.5abs LUR models 2-week measurements in three different seasons (warm, cold, and intermediate) from monitors at site between Feb 2009 and Feb 2010	**Exposure to NO**_**x**_ **from 0–2 and 2–5 years** ↑ rsFC in areas in auditory association, premotor, orbital and polar frontal, inferior parietal, and posterior cingulate cortices, in the ventral diencephalon, and in the MT+ complex and neighbouring visual areas **Exposure to NO2 during pregnancy and from 0–2 years** ↑ rsFC in areas in in auditory association, ventral diencephalon, and insular and frontal cortices opercular, somatosensory and motor and early auditory, dorsal stream visual and superior parietal **Exposure to PMcoarse from 2–5 and 5–9 years** ↑ rsFC in areas in anterior cingulate and medial prefrontal cortices and in the MT+ complex and neighbouring visual areas **Exposure to PM2.5abs from 0–2 and 2–5 years** ↑ rsFC in areas in insular and frontal opercular, auditory association, lateral temporal, somatosensory and motor, anterior cingulate and medial prefrontal, and posterior cingulate cortices, and in the MT+ complex and neighbouring visual areas	[22] Generation R study n = 2197
Prenatal and childhood	Whole pregnancy and childhood at periods: 0–3 years 3–6 years 6 – age of MRI	Preadolescence 9–12 years	NOx, NO2, PM10, PM2.5, PM2.5abs LUR models 2-week measurements in three different seasons (warm, cold, and intermediate) from monitors at site between Feb 2009 and Feb 2010	**Exposure to NOx from 3–6 years** ↑ rsFC in regions of the visual and task positive networks: MT+ complex and neighbouring visual areas—inferior frontal cortex and MT+ complex and neighbouring visual areas—insular and frontal opercular cortex **Exposure to NO2 from 0–3 years** ↑ rsFC in regions of the visual, auditory and task positive networks: dorsal stream visual cortex – superior parietal cortex and auditory association cortex – insular and frontal opercular cortex **Exposure to PM2.5abs from 0–3 years** ↑ rsFC between brain regions of several networks (19 of 22): visual - visual, visual – auditory, visual – task positive, visual – task negative, auditory – task positive, auditory – task negative, and task negative – task negative ↓ rsFC between brain regions of visual – task positive networks and task positive – task negative networks (3 of 22): MT + complex and neighbouring visual areas – superior parietal cortex, posterior cingulate cortex – superior parietal cortex, and frontal opercular cortex – lateral temporal cortex	[79] Generation R study n = 2197
Prenatal and childhood	48-h during last trimester 5 years of age	Childhood Mean age ± SD: 8.0 ± 1.3 years	PAH Personal air monitors during last trimester Urine samples in childhood	**Prenatal exposure to PAH** ↓ local volume in the middle frontal gyrus, medial orbitofrontal gyrus, inferior frontal gyrus, superior frontal gyrus, pre-central gyrus, post-central gyrus, supramarginal gyrus, middle temporal gyrus, superior temporal gyrus, mesial superior parietal gyrus, praecuneus, cuneus, cingulate gyrus, gyrus rectus in the left hemisphere. ↓ WM surface extending throughout the left hemisphere. No association with cortical thickness **Postnatal exposure to PAH** ↓ WM surface in dorsolateral prefrontal regions, especially over the superior frontal gyri	[26] CCCEH study n = 40
Lifetime	From birth (2001–2003) to 12 years of age	Childhood 12 years	EC (high vs low exposure group) LUR models Measurements from 27 monitors at site between 2001 and 2006, and simultaneous 24-hour sampling at 4–5 sites over different seasons	↓ CT in the medial frontal gyrus, ventromedial prefrontal cortex (R), paracentral lobule, postcentral gyrus, superior frontal gyrus, precentral gyrus (L), inferior parietal lobule (L), superior parietal lobule (L), anterior cingulate (L), cingulate (R), precuneus (L), fusiform gyrus (L) ↓ GM volume in the cerebellum, precentral gyrus, inferior parietal lobule	[28] CCAAPS study n = 135
Adulthood (1998–2001)	1 year (2000)	Adulthood (1999–2005) ≥ 60 years Median age [IQR]: 68.0 [9.0] years	PM2.5 Spatiotemporal model	↓ total cerebral brain volume No association with hippocampal volume	[40] Framingham Offspring Study n = 943
Adulthood (2006–2010)	1 year (2010)	Adulthood (2014) 44–80 years	NOx, NO2, PM10, PMcoarse, PM2.5 LUR models	↓ total GM volume with higher exposure to any of the investigated pollutants ↓ GM volume in the frontal pole and operculum cortex (L) with higher exposure to PM10 ↓ GM volume in the frontal pole, operculum cortex (L), and orbital cortex (R) with higher exposure to NOx ↓ GM volume in the frontal pole (R) and operculum cortex (L) with higher exposure to NO2 **Exposure to PM2.5** ↓ total WM volume ↓ GM volume in the frontal pole, orbital cortex (R), operculum cortex (L) **Exposure to PMcoarse** ↓ GM volume in the frontal pole (R), superior gyrus (L), operculum cortex (L) ↓ volume in the thalamus (L)	[41, 49, 51] UK Biobank n ≈ 18,290
Adulthood (1999–2005-6)	3, 8, and 10 years (1999–2005-6)	Adulthood (2005–2006) 71–89 years	NO2, PM2.5, diesel PM Spatiotemporal model	**3-year cumulative exposure to NO2** ↓ GM volumes in the prefrontal cortex ↓ volumes in the anterior cingulate gyrus, insula, amygdala, limbic medial temporal lobe, basal ganglia **3-year cumulative exposure to PM2.5** ↓ WM volumes in the anterior and posterior extreme/ external capsule, calcarine gyri ↓ GM volumes in the superior, middle, medial frontal gyri, inferior frontal gyrus (L), superior parietal lobule, occipital poles ↓ volume in the anterior cingulate gyrus ↑ volumes in the thalamus, putamen, globus pallidus, posterior insula No association with volumes of corpus callosum, hippocampus, temporal lobe **8-year cumulative exposure to PM2.5** ↓ total WM volume and in the frontal, parietal, temporal lobes, corpus callosum No association with hippocampal, basal ganglia volumes and GM volumes across the cerebral cortex **10-year cumulative exposure to diesel PM** ↑ ventricular volume **U**-shaped associations were observed for total WM volumes and in frontal, parietal and temporal lobes ↓ total GM volumes and in frontal, parietal and temporal lobes	[42–44, 50, 52] WHIMS n = 764, 1403, 1365 n = 1403 (8- and 10-year assessment)
Adulthood (1990–1998, 1999–2007, and 1990–2007)	5–20 years assessed at three 8-year periods (1990–1998, 1999–2007, and 1990–2007)	Adulthood (2011–2013) Mean age ±SD: 76.0 ±5.0 years	PM10, PM2.5 Spatiotemporal model	↓ deep-GM volumes ↓ volumes in total brain, frontal and parietal lobe in one of the study centres with higher exposure to PM2.5 No association with hippocampal volume	[45] ARIC study n = 1753
Adulthood (2006–2008) 50–80 years	2 years (2006–2008)	Adulthood (2011–2015) 55–85 years	NOx, NO2, PM10, PM2.5, PM2.5abs LUR models Three separate 2-week periods (to cover different seasons) between Oct 2008 and Oct 2009	Local atrophy in inferior parietal lobule (R) with higher exposure to NOx, PM10, and PM2.5 Local atrophy in posterior cingulate cortex and praecuneus (R) with higher exposure to NOx, NO2, and PM10 No association with local atrophy in the dorsolateral prefrontal cortex	[46] 1000BRAINS n ≈ 615
Adulthood (2000–2003) Mean age: 56.1 years	3 years (2000–2003)	Adulthood (2011–2015) 56–85 years Mean age: 67.4 years	NO2, PM10, PM2.5, PM2.5abs LUR models Three separate 2-week periods (to cover different seasons) between Oct 2008 and Oct 2009	↓ Intra-network rsFC and segregation index in the dorsal attention network with higher exposure to NO2 ↓ Intra-network rsFC in the ventral attention network with higher exposure to PM10 and PM2.5 ↑ Inter-network rsFC in the visual network with higher exposure to PM2.5abs ↓ segregation index in the ventral attention network with higher exposure to PM2.5abs	[54] 1000BRAINS n = 574
Adulthood (2015–2019)	1 year	Adulthood (2015–2019) Mean age ±SD: 49.5 ± 13.3 years	NO2, PM2.5, ozone Hybrid kriging-LUR models	↑ volume in rostral middle frontal (L), supramarginal (L), transverse temporal (L), pars opecularis (R) with higher exposure to NO2 ↑ volume in pars triangularis (L) and CT in fusiform (R) with higher exposure to PM2.5 ↑ pars orbitalis volume (L) with higher exposure to ozone No association with WM and GM volumes	[47] Taiwanese sleep study n = 4866
Adulthood (2014–2017)	5 years prior to the recruitment intervals (NO2, PM10) (2010–2014, 2011–2015, and 2012–2016) 1 year (PM2.5) (2015)	Adulthood (2014–2017) Mean age ±SD: 67.3 ± 6.4 years	NO2, PM10, PM2.5, PAH metabolites Kriging model Annual concentrations of PM10 and NO2 at monitoring sites between 2001 and 2016. Same method used for PM2.5 Urine samples (PAH)	**Exposure to NO2** ↓ volume in caudate, pallidum, amygdala, nucleus accumbens ↓ CT in the frontal cortex, lateral temporal cortex, inferior parietal cortex, posterior cingulate, insula, parahippocampal gyri, fusiform gyri ↑ CT occipital cortex, postcentral gyri (L) **Exposure to PM10** ↓ volume in pallidum, putamen, amygdala, nucleus accumbens ↓ CT in the lateral temporal cortex, inferior parietal cortex, prefrontal cortex, posterior cingulate, insula, parahippocampal gyri, fusiform gyri ↑ CT occipital cortex, postcentral gyri **Exposure to PM2.5** ↓ volume in nucleus accumbens ↓ CT in the lateral temporal cortex, inferior parietal cortex, prefrontal cortex, insula, parahippocampal gyri, fusiform gyri ↑ CT occipital cortex, postcentral gyri **Exposure to PAH (highest vs lowest quartile)** ↓ CT in parietal, temporal and insular lobes in men ↓ CT in frontal and parietal lobes in women ↓ volumes in the caudate in men, and pallidum in women	[48, 252] EPINEF study n = 957 n = 528 (PAH)
Adulthood (2016)	1 year (2015)	Adulthood (2016) 20–48 years	NO2, NOx, PM10, and PM2.2 Berlin’s Senate Department for Urban Development and Housing	**Exposure to NO2 and NOx** No association was observed **Exposure to PM2.5 and PM10** ↓ stress-related activation in frontoinsular cortex, hippocampus, amygdala, ventral striatum, inferior parietal cortex, thalamus, precuneus, posterior cingulate cortex, anterior cingulate cortex, dorsolateral, ventrolateral, and ventromedial prefrontal cortex. The associations were more pronounced for PM2.5 than PM10	[56] Berlin neuroimaging study n = 42 men
Adulthood	120 min	Adulthood Mean age ±SD: 27.4 ±5.5 years	Diesel exhaust (intervention arm) Filtered air (control arm)	No differences in the default mode network rsFC for post- compared to pre-diesel exhaust exposure ↑ rsFC in the middle temporal gyrus (R), occipital fusiform gyrus (R) for post-filtered air compared to pre-filtered air ↑ rsFC in the angular gyrus (R), frontal pole, middle frontal gyrus, middle temporal gyrus, praecuneus cortex, temporal pole (L) for post-filtered air compared to post-diese; exhaust	[253] Randomised cross-over study n = 25

*ABCD* Adolescent Brain Cognitive Development, *ADC* average diffusivity coefficient, *ARIC* Atherosclerosis Risk in Communities, *BPA* benzo[a]pyrene, *CCAAPS* Cincinnati Childhood Allergy and Air Pollution Study, *CCCEH* Centre for Climate Change and Environmental Health, *CT* cortical thickness, *EC* elemental carbon, *EPINEF* Environmental Pollution-Induced Neurological EFfects, *FA* fractional anisotropy, *FC* functional connectivity, *GM* grey matter, *L* left, *LUR* land-use regression, *MD* mean diffusivity, *MRI* magnetic resonance imaging, *NO*_*x*_ nitrogen oxides, *NO*_*2*_ nitrogen dioxide, *OC* organic carbon, *OP* oxidative potential of PM2.5, *PAH* polycyclic aromatic hydrocarbons, *PM* particulate matter, *PM2.5* particulate matter with aerodynamic diameter ≤2.5 μm, *PM2.5abs* absorbance of the PM2.5 fraction, *PM10* particulate matter with aerodynamic diameter ≤10 μm, *PMcoarse* particulate matter with aerodynamic diameter between 10 μm and 2.5 μm, *R* right, *rN0* restricted isotropic intracellular diffusion, rs resting state, *SA* surface area, *WHIMS-MRI* Women’s Health Initiative Memory Study-MRI, *WM* white matter.

Prenatal exposure to fine PM (PM2.5) and PAH was associated with smaller white matter (WM) volume in parietal lobes [27], and WM surface reductions in the left hemisphere in later childhood, mediating the association between air pollutants and conduct disorder problems [26]. Furthermore, early life exposure to TRAP was associated with increased frontotemporal cortical thickness in children and adolescents [22, 27, 28]. Alterations in global WM microstructure and in several WM microstructure tracts were documented [22, 27, 30, 32]. Hemispheric asymmetry in WM and grey matter (GM) volumes across all cortical regions and several subcortical regions has been observed [30, 33–36] (Table 1). While brain asymmetry is a typical trait in humans, it can be altered and has been linked to psychiatric disorders [37–39].

Air pollution’s influence on brain health may extend beyond early brain development, including later stages of life, as suggested by cross-sectional and longitudinal assessments (Table 1). Among adults exposed to different components of air pollution, studies have reported volume reductions in total cerebral brain [40], total WM and GM [41–44], deep-GM [45] and local atrophy mainly in frontocortical areas, insula and subcortical regions [46–53]. These changes partially mediated the association between PM2.5 and NO2 with depressive symptoms [52]. Functional neuroimaging studies, including a 10-year prospective study [54] and a 6-month assessment of air pollution [55], showed reduced stress-related activation in connectivity networks associated with acute stress, such as the salience, DMN, and central executive networks, in adults with higher exposure to air pollution [54], and augmented stress-related information transfer across cortical and subcortical brain networks among participants with a higher polygenic risk score for depression [55]. Furthermore, a negative stress-related brain activation was observed in prefrontal cortex (PFC), frontoinsular cortex, limbic system, inferior parietal cortex, praecuneus, and cingulate among men living in residential areas with higher PM2.5 and PM10 levels [56]. Nevertheless, opposite activation directions than expected were observed in the amygdala that could not be explained with certainty. Altogether, these findings suggest that air pollution may increase vulnerability to mood dysfunction and potentially inhibit an appropriate stress response.

Taken together, current literature indicates that exposure to air pollution may have diverse and hemisphere-specific implications on brain morphology and function in children and adults (Table 1). Air pollution effects on brain regions appear to vary depending on the specific pollutant and period of assessment during the lifespan. It is important to note that while longitudinal studies assessed air pollution exposure prior to outcome measurement, they are constrained by the lack of information regarding the timing of changes in brain morphology and function during the follow-up period, as only one MRI assessment was conducted. Although concrete conclusions cannot be made, disruptions were observed in regions such as PFC, anterior cingulate cortex (ACC), hippocampus, amygdala, insula, NAc, corpus callosum and striatum, all of which have been implicated in the risk for major psychiatric disorders [57, 58], like depression, anxiety [59–62], substance use disorders [63, 64] and schizophrenia [57, 65].

Epidemiological studies have provided evidence linking air pollution to mental health disorders in exposed youth and adults [66]. Recent meta-analyses of observational studies highlighted a positive association between PM2.5, PM10 and NO2 exposure with risk for depression [67] and suicide [68]. Furthermore, available evidence suggests that short- and long-term exposure to PM2.5 is linked to an increased risk for anxiety, while exposure to PM10, NO2, and NOx might increase the risk for schizophrenia or hospitalisation for schizophrenia [69, 70]. By linking epidemiological approaches on air pollution with neuroimaging data, future studies can help elucidate mechanisms by which air pollution-induced neuroinflammation and other potential biological pathways, such as stress response [17] may affect brain, behaviour, and psychopathology.

## Noise pollution

Noise pollution originates from urban traffic, airports, industries, and construction sites and can evoke negative emotions and annoyance. Prolonged exposure to disruptive noise is thought to induce brain alterations through mechanisms such as sleep disturbances, which prompt a pro-oxidative environment, predisposing to neuroinflammation, and heightened hypothalamic-pituitary-adrenal (HPA) axis reactivity [71, 72], that might contribute to mental illness [73, 74]. Residents in a community impacted by changed flight patterns compared to a demographically similar non-impacted community, showed a higher risk for substance use and mental health-related emergency visits among individuals living in noise-affected communities, particularly in younger age groups [75]. Meta-analyses have reported increased odds for depression and anxiety with higher 24-h noise level [76]. Still, the association between noise and mental health is limited due to high risk-of-bias studies and inconsistent findings across studies included in the different systematic reviews [76–78].

The relationship between noise pollution and brain structure and function remains understudied and is also afflicted with inconsistent findings [79, 80]. A study on 8–12-year-olds exposed to school road-traffic noise over one year reported enhanced connectivity in the subcortical auditory pathway [81], indicating possible enhancement on auditory processing abilities but also increased sound sensitivity and sensory overload. Whether these results, along with potential noise-induced chronic stress and sleep disturbance contribute to anxiety and behavioural problems in children requires further investigations. Among older adults participating in a 5-year study, higher noise pollution was associated with cognitive decline and alterations in brain network organisation were reported [46, 54]. A small cross-sectional neuroimaging study involving healthy men did not find an association between noise pollution at the place of residence and neurofunctional activation during a social-stress paradigm [56]. Further studies on the behavioural and cognitive consequences of noise pollution across the lifespan are required to provide robust evidence and establish explicit mediating brain structures and functions.

## Light pollution

Light pollution, a consequence of human activities, including outdoor lighting, commercial signage, and illuminated buildings, produces excessive or misdirected artificial light and disrupts the natural darkness of the night sky. Exposure to artificial light at night (ALAN) has become increasingly prevalent, especially in urban areas. Light is detected by the retina and transmitted through the intrinsically photosensitive retinal ganglion cells (ipRGCs) to the suprachiasmatic nucleus in the hypothalamus and other brain regions involved in regulating circadian rhythms and sleep-wake cycles [82]. Circadian rhythm disruptions have been linked to an elevated risk of major depressive disorders, bipolar disorders, and heightened mood instability [83], potentially mediated by oscillations in clock genes expression responsive to light-dark transitions [84]. Light is also projected (via the ipRGCs and the suprachiasmatic nucleus) to regions involved in mood regulation, such as the PFC, hippocampus, and amygdala [85, 86], directly influencing emotional processing and mood functions [87, 88]. Hence, prolonged and ill-timed ALAN exposures may precipitate or worsen symptoms of mood disorders.

Cross-sectional analyses reported an increased prevalence of mood and anxiety disorders in adults and adolescents with higher exposure to outdoor ALAN [89–91]. However, residual confounding due to air pollution has likely influenced the results [91]. We found no studies examining the relationship between ALAN and brain structure and function. Participants exposed to dim ALAN during one-night sleep in a polysomnography laboratory exhibited decreased brain activity in the inferior frontal gyrus (IFG) compared to a night without any light exposure [92]. Decreased activation in the IFG has been associated with impairments in executive functions and are reported in clinical populations afflicted with bipolar disorder, depression, and schizophrenia [93–96]. Still, further research is needed to elucidate the effects of light pollution on brain changes.

## Urbanisation

Urbanisation is a shared element in global migration patterns over the past half-century, involving the transition from rural to urban settlements [4]. Historically, this transition has been linked to economic growth. Urban dwellers are more likely to benefit from sustainable infrastructure, essential education, healthcare services, and more work opportunities than rural residents. Despite these advantages, the urban environment is inhomogeneous, depicted by economic, social, and environmental inequalities [4, 97]. Rapid and unplanned urbanisation increases income inequalities, linked to disparities in health and education, marginalisation, social isolation and threat, and environmental pollutants [97–99]. Within this context the urban environment has been associated with mental disorders, such as depression, anxiety and schizophrenia [99–104]. Urban upbringing has been identified as the most prominent risk factor for schizophrenia [9, 102], possibly stemming from social characteristics of the urban environment, including decreased social capital and cohesion, social deprivation and fragmentation, and affecting neural mechanisms of social stress processing and positive social interactions [105, 106].

A common underlying mechanism linking urban living stressors to vulnerability to mental illness has been suggested to be the dysregulation of the HPA-axis [17, 97, 107, 108], potentially resulting in cerebral functional and structural changes [109]. Moreover, urban environments may interact with genetic variations in genes related to stress response and brain structure, such as neurodegeneration, neural differentiation, and axon growth [110, 111]. Various neuroimaging studies reported the association between urban scenery image viewing and functional changes in brain regions implicated in emotional and stress responses [112–114].

Current city living and urban upbringing were associated with increased activity in the amygdala-hippocampus complex and subgenual ACC, respectively, during a stress task in small neuroimaging studies of healthy adults [105, 111], suggesting dysregulation in stress processing. Furthermore, a cross-sectional study among older healthy adults living in areas with higher percentage of urban landscape reported a negative association with GM volume in the perigenual/subgenual ACC, association which remained significant after adjustment for residential greenness and other confounders. Contrasting effects on the same brain region were observed with higher percentage of urban green [115]. The ACC is a key region for regulating amygdala function, negative emotions, and stress and has been proposed to mediate the relationship between medial PFC (mPFC) activity and affective symptoms [116]. Among urban dwellers a dysregulation of mesolimbic dopamine system during the desire-reason-dilemma paradigm [117] has been observed, through which urban environment may be further linked to an elevated the risk for mental disorders, including schizophrenia and depression [118].

Urban environment during childhood may alter amygdala and PFC activation in adulthood by interacting with genes related to dopamine, anxiety, and stress phenotypes, whereas such effects were not evident among individuals with a rural or small-town upbringing [119, 120]. Urban upbringing was associated with reduced hippocampal and amygdala volumes among adolescents [121] and GM dorsolateral PFC (dlPFC) and mPFC in adults [122–124]. Healthy adults with higher urban upbringing scores were observed to have cortical thinning in the dorsolateral, mPFC, and parahippocampal cortex [125], although findings are not consistent [126]. Stress-induced volume reductions in the observed regions during childhood have been associated with depression, psychosis, and post-traumatic stress disorders in later life [127–129], while the identified cortical thinning aligns with regions implicated in psychiatric conditions, including schizophrenia and bipolar disorders [130]. To what extent brain changes in these disorders are driven by urbanicity remains to be determined.

The urban environment encompasses various economic, social, ambient, and infrastructural characteristics. Current literature assessed urban living based on a measure of population density and duration of residency (described in this section) or by using isolated factors, such as pollution, urban green spaces, and socioeconomic deprivation, which often co-occur and interact within individuals’ living environment. A study using a composite measure of urban living, including night-time lights, green space, build-up space, water bodies and land use, reported an association with certain changes in brain structure and function. These changes included a reduced mPFC volume, increased cerebellum volume, and decreased functional network connectivity within the mPFC of the anterior DMN. The findings were consistent across the two cohorts of young adults residing in Europe and China, as observed in cross-sectional assessments. The observed neural correlates mediated the association between urban living and depressive symptoms [131]. In addition, cross-sectional analyses on a comprehensive set of factors related to urban living identified environmental profiles relevant to psychiatric symptoms in a large cohort of adults living in the UK. In particular, an environmental profile predominantly characterised by regional deprivation, pollution and density of urban infrastructure was positively associated with affective symptoms and mediated by smaller striatum volumes, while an environment characterised by dense build-up space and mixed land use was associated with anxiety symptoms and was mediated by reduced volumes of IFG, amygdala and cerebellar regions. The associations were moderated by genes related to stress response regulation, anxiety, and phobia, suggesting that genetic variations may explain individual differences in response to environmental adversity [110].

Further research is warranted that accounts for the inherent complexity of the living environment to disentangle the distinct and interconnected attributes of urban environments that contribute to brain function and dysfunction.

## Natural space

Two prominent frameworks have been suggested to explain the effects of natural environments, such as surrounding green spaces, forests, or water bodies on mental well-being. The attention restoration theory (ART) posits that nature facilitates the restoration of attentional capacity, reduces mental fatigue, and enhances focus and cognitive functioning, ultimately contributing to improved mental well-being [113, 132–134]. Simultaneously, the stress reduction theory (SRT) proposes that nature lowers physiological and psychological stress and enhances positive feelings [135, 136]. These effects occur via mechanisms involving the autonomic nervous system, reflected by lower blood pressure and improved heart rate, as well as the modulation of the endocrine system, including reductions in stress hormone secretion [137, 138]. While both theories acknowledge the beneficial effects of nature, ART emphasises the cognitive benefits, particularly regarding attention restoration, while SRT emphasises the emotional benefits, particularly stress reduction.

Nature-induced benefits on the central nervous system have been observed in experimental, intervention and observational studies, corroborating the notion that contact with nature promotes mental health. Compared to urban scenes, viewing natural landscapes in a laboratory setting was linked with cognitive restoration, reduced visual attention focus [132], and activation of brain areas associated with positive emotional responses, rewarding experience, and recollection of positive memories [112, 139–141]. Additionally, nature images evoked enhanced FC between the DMN, dorsal attention network, ventral attention network, and the somatomotor network, potentially promoting cognitive coherence and effortless attentional engagement [142].

A brief walk in nature showed positive effects on brain and mental health by decreasing PFC activation, which is associated with sadness and behavioural withdrawal, and reducing rumination—a pattern linked to depression [143], possibly via the restorative benefits of nature. Additionally, after a nature walk, there was a decreased amygdala activation during a social stress-inducing task, a region responding to fear and stress [144]. Such benefits were not observed following urban walks [143, 144]. Nevertheless, sex-specific differences in response to natural and urban environments warrant further investigation [145]. Surrounding urban green space, assessed using location tracking for a week [146] or governmental databases [56], also seemed to have supportive effects on coping with stress (aligning with the stress reduction theory), as indicated by activation patterns in emotion-regulatory brain areas, like the dlPFC, mPFC, insula, ACC, posterior cingulate, and ventral striatum [56, 146]. Here again, opposite activation directions than expected were observed in the amygdala [56].

Higher residential greenness was further associated with morphological brain changes in cross-sectional [110, 147–149] and longitudinal studies [150, 151]. The findings encompassed higher GM and WM volumes in the PFC, premotor cortex, and cerebellum in children [151], and lower global atrophy, higher GM orbitofrontal cortical volume, and thicker PFC, insula and praecuneus in adults [147–150]—structures linked to cognitive process and psychiatric disorders when reduced [152–156]. Indeed, reduced volumes in frontolimbic and cerebellar regions were observed in environments characterised by reduced access to natural spaces that mediated the association between urban living and affective and anxiety symptoms [110].

Further research is needed regarding the different typologies of natural spaces and vegetation, which is currently lacking. For example, among older adults, only neighbourhood forest exposure seemed to positively affect amygdala integrity, but not urban green or blue spaces [157]. The presence of green space in the living environment was associated with reduced risk of depression and anxiety in cross-sectional studies [158], however, non-consistently, and similar associations were not supported by longitudinal studies [101, 159]. These discrepancies possibly arose due to methodological shortcomings, such as an inability to assess whether participants spent time in those environments, and the mediating effects of air and noise pollution or exercise uptake. Different buffer areas, which are specific zones established around the location of participants to indicate distance, were used in the literature. Yet, it remains unclear which zone size is the most relevant for mental wellbeing. Furthermore, distance to green areas was typically calculated with Euclidean distance, which calculates the straight-line distance between two points, rather than pathways people use or road connections. This approach might not accurately reflect the experiences of the local urban population.

## Regional socioeconomic status

Regional socioeconomic status can significantly influence the cognitive, emotional, and behavioural development of children and adolescents, and these effects may persist in adulthood [160–163]. Youth growing up in disadvantaged neighbourhoods, marked by poverty, violence, poor housing conditions, or limited access to educational and healthcare resources [164], are often exposed to higher levels of chronic stress and unpredictability [165], and may have difficulties building supportive social networks [166]. Consequently, they may face a higher risk of childhood mental disorders [167, 168]. Similarly, living in deprived neighbourhoods during adulthood has been associated with pooled mental health disorders, depression, suicidal behaviour, and self-harm [101, 169–171].

Neighbourhood disadvantage has been linked to HPA-axis dysregulation and reactivity [172, 173]. Additionally, cross-sectional studies in youth have shown alterations in neural development and functioning related to cognitive processes, rewards, and social threats. For instance, lower neighbourhood socioeconomic status associated with decreased activation in regions of the executive system, including the dlPFC, posterior parietal cortex, praecuneus and cerebellum, during a working memory task [174]. Neighbourhood poverty may also disrupt self-control development, measured with inhibition performance task, via its effect on IFG activation [175]. Furthermore, youth living in more deprived areas recorded lower activation in caudate, putamen, accumbens area and pallidum during reward anticipation [176], and higher amygdala reactivity to threat-related stimuli, particularly in neighbourhoods where safety and management norms were more permissive [177]. Altogether, these responses have been implicated in internalising and externalising symptoms and psychopathology [178–180].

Cross-sectional studies have further indicated changes in connectome in youth residing in socioeconomically disadvantaged areas suggest that neighbourhood deprivation impede the developmental progression of brain function in children and young adults [181], involving reduced fronto-amygdala and fronto-striatal resting state FC [182–184], and changes in FC between DMN and dorsal attention network and sensorimotor systems [185]. The observed connectome alterations were coupled with internalising symptoms and worse cognition. Furthermore, increased fronto-striatal FC in newborns living in deprived neighbourhoods mediated the relationship between disadvantage and externalising symptoms at age 2 years [186].

Compared to the above findings, different patterns in FC were observed when community violence and crime were assessed. Such experiences associated with FC changes in youth and newborns (exposed prenatally) between regions of the limbic system, mainly encompassing the hippocampus [187, 188]. Furthermore, youth exposed to community violence demonstrated FC changes between the hippocampus and insula, with opposing directions observed across studies [187, 189]. These discrepancies may be influenced by various factors, including the specific timing of exposure to community violence, developmental changes, individual characteristics, or other contextual factors, such as positive parenting and school environment [185, 190, 191]. Differential social experiences, such as discrimination, within similar environments may exert distinct neural influences on minoritized and discriminated individuals, including various racial and gender identities, particularly in the domains of threat, reward and emotional processes [165, 192–194].

Neighbourhood adversity in adolescence may shape neural responses to social situations, threats, and rewards in adulthood. Individuals with a disadvantaged upbringing displayed increased sensitivity in reward-related brain regions like the striatum, NAc, and ventrolateral PFC. Notably, current income did not mediate the observed associations, suggesting a potential link between early experiences and reward anticipation and pursuit in later life [195]. Furthermore, exposure to neighbourhood disadvantage during adolescence might influence reward-related processes in adulthood, via decreased activation in brain regions associated with cognitive and affective processes, such as amygdala, hippocampal and dlPFC [196]. Lastly, neighbourhood quality might influence neural responses to social stimuli, as observed by increased activity in the dorsal ACC and prefrontal regions among individuals with disadvantaged upbringing [197].

Several studies have demonstrated the effect of neighbourhood disadvantage on brain structure in youth and adults, such as widespread lower volume of WM and GM [198, 199], including hippocampus [189, 199–201], amygdala [189], dlPFC and dorsomedial PFC, superior frontal gyrus [201], IFG and ACC [202]. In addition, smaller surface area and cortical thinning was observed in the frontal, parietal, and temporal lobes, cingulate and insula [203–207]. The majority of these studies employed a cross-sectional design, with two exceptions [189, 205]. Finally, neighbourhood disadvantage was linked to atypical neurodevelopmental trajectories during adolescence, indicating delayed brain development [208, 209]. It is currently unknown whether the deviations in brain trajectories due to adversity, converge later in development or if they reflect atypical developmental patterns.

Altogether, neighbourhood disadvantage was associated with alterations in brain regions involved in emotional processes, including the amygdala, hippocampus, and dlPFC, and reward-related regions such as the striatum and NAc. Several studies accounted for individual or family socioeconomic status as a confounding variable, demonstrating that regional socioeconomic status may exert distinct effects on brain and behaviour. Most studies evaluated neighbourhood disadvantage as a single measure of neighbourhood violence or poverty, or used a composite score structured from several measures (e.g., poverty, unemployment rate, education levels). However, assessing different attributes of regional challenges might elucidate distinct neural correlates to different adversity typologies [210].

## Weather patterns and climate change

Weather patterns encompass various meteorological factors, including temperature, precipitation, humidity, and sunlight duration. Mounting evidence suggests that weather patterns may influence mood, behaviour, and overall mental well-being. Higher ambient temperatures have been associated with an increased suicide or self-harm burden [68, 211, 212], mental health-related mortality, and morbidity of schizophrenia, mood disorders, and anxiety disorders [213, 214]. Likewise, higher humidity has been linked with a greater burden of concurrent depression and anxiety, increased mental health-related emergency visits [214, 215], and aggravation of the adverse effects of high temperatures [216]. Regarding precipitation patterns, limited evidence suggests a possible positive link with mental illness [217–219]. Studies have reported a negative association between sunlight exposure and risk of depression and anxiety [158], while cloudiness and decreased sunshine duration were linked to increased suicide rates [220]. Furthermore, seasonal changes directly affect the duration of daylight. Seasonal daylength fluctuations appear to affect mood and behaviour negatively and were associated with a higher prevalence of seasonal affective disorder and earlier onset of bipolar disorder [221]. Here it is important to acknowledge that many of these findings are susceptible to bias due to inadequate control of confounders and the risk of an ecological fallacy—the incorrect inference about individuals based on aggregated-level data associations [222].

The changes in weather patterns associated with climate change introduce new challenges that further complicate mental health outcomes via direct effects of stress and trauma and indirect mediating factors, including food insecurity, poverty, climate change-induced violence and forced migration [104]. Extreme weather events include heatwaves, flooding, and drought. Systematic investigations demonstrated positive associations between heatwaves and mental health-related morbidity [213, 223], where greater frequency, duration, and intensity of heatwave conditions appeared to magnify the observed effects [213, 224]. Direct exposure to floods was associated with depression, anxiety, post-traumatic stress disorder, suicidal ideation, and psychological distress [214, 225, 226]. Similarly, droughts were associated with increased psychological distress, especially among rural inhabitants and vulnerable populations [227, 228]. The neural circuits linking weather and psychiatric risk are unclear, as studies investigating the weather and climate change effects on MRI-detected brain activity are lacking. During simulated hyperthermia conditions (50 °C, >40 min), there was heightened activation in the dlPFC and the right intra-parietal sulcus [229]. Additionally, impairments in the FC of the DMN were observed [230], coupled with prolonged reaction time in cognitive tasks compared with the control group [229, 230]. A few cross-sectional studies reported positive associations between day length and volumes of the hippocampus, amygdala, and brainstem—regions that exhibited seasonal variations in serotonin signalling [221], suggesting that changes in volumes of subcortical regions and neurotransmitter signalling involved in emotional regulation may be involved in the seasonality of mental disorders.

## Perspective

The existing literature suggests potential associations between the macroenvironment and the physiological development and ageing of the brain. However, reaching definitive conclusions is challenging due to the limitations in the study designs, which prevent the establishment of causal inferences or temporal patterns. Furthermore, current findings are either contradicting or lack specificity, as multiple regions show an association with macroenvironmental adversity, particularly in relation to air pollution. These observations may result from the diverse selection of regions of interest, the timing and severity of exposure. The influences of macroenvironmental adversity on the brain may be more immediate or manifest over time, depending on the specific exposure and brain region [21, 131], while the reversibility of unfavourable changes in structure and function following exposure to factors that contribute to resilience is unclear [231].

Research investigating the associations of light and noise pollution, weather patterns and extremes on the brain is notably limited. Certain brain regions have been consistently reported to show changes in response to the other macroenvironmental factors. The common brain areas include regions involved in emotional regulation, such as PFC, amygdala, hippocampus, and ACC, similar to the effects observed in microenvironmental adversity [232], as well as regions related to reward processing, such as striatum and NAc. More specifically, urbanicity, air pollution, and regional deprivation demonstrated unfavourable effects on these brain regions, while natural spaces were associated with beneficial effects. Furthermore, distinct neural regions have also been associated with different types of environmental adversity. For example, current city living was associated with amygdala activity, while urban upbringing affected ACC [105]. Similarly, neighbourhood poverty appeared to impact FC between PFC, amygdala, and striatum, while changes in FC mainly involving the hippocampus were observed with exposure to neighbourhood violence.

Environmental influences on the brain can contribute to inter individual differences in mental health, shaping susceptibility to conditions like depression, anxiety, and cognitive impairments. The brain, as a central mediator, plays a pivotal role in processing environmental stimuli and translating them into neurobiological responses. It acts as a bridge between macroenvironmental exposures and mental health outcomes. Neuroplasticity, the brain’s ability to adapt and reorganise in response to experiences, may be a key mechanism through which environmental factors leave lasting imprints on mental health. Moreover, the brain’s intricate network of neurotransmitters, hormones, and neural pathways can modulate emotional states and stress responses, contributing to mental well-being or susceptibility to mental disorders. Mental health reflects the cumulative impact of these environmental and neural processes. It encompasses a spectrum of emotional, cognitive, and behavioural outcomes, ranging from resilience and well-being to the manifestation of psychiatric disorders. This speculative framework highlights the interplay of environmental, neural, and psychological factors. However, the brain may also play a role in the selection of specific environments, which may be partly driven by genetic factors [233]. The need for comprehensive research to better understand the diverse pathways involved should be emphasised once again.

Environmental factors may exert their influence on the brain through shared or different pathophysiological mechanisms. Often these mechanisms are interrelated, that one is not considered separately from another. Table 2 provides an overview of the contributing factors postulated to mediate the effects of the macroenvironment on brain health. Nevertheless, the list is not exhaustive, and comprehensive reviews can be found elsewhere [5, 17, 71, 82, 108, 221, 234]. More mechanistic studies are needed to elucidate the underlying pathways for the differential links of various macroenvironmental factors with specific brain regions, despite eliciting common effects, such as HPA-axis activation and neuroinflammation.

**Table 2:** Putative pathophysiological mechanisms involved in the association between macroenvironmental factors and brain health.

Mechanism/ Pathway	Associated Macroenvironmental Factors
Inflammation	Air Pollution, Noise Pollution, Heat exposure
Hypothalamic-pituitary-adrenal-axis dysregulation/ Stress response	Noise pollution, Urbanisation, Regional Socioeconomic Factors, Natural spaces
Oxidative stress	Air pollution, Noise pollution, Heat exposure
Circadian rhythm disturbances/ Sleep disturbances	Light Pollution, Noise pollution, Seasonal daylength fluctuations
Attention Restoration	Natural spaces

## Future research directions

### Overcoming the complexity of high dimensional data

The associations between the macroenvironment, brain outcomes and mental health involve complex interactions between multiple environmental exposures, individual susceptibility and social factors. As individuals are typically exposed to multiple stressors simultaneously, it becomes challenging to quantify the impact of a specific environmental factor. Furthermore, high correlations are usually present among the different environmental factors adding complexity in determining their independent effects. High collinearity might lead to unstable or imprecise coefficient estimates [235]. Indeed, most studies have primarily focused on exploring the relationships between a singular exposure and brain outcomes or mental health, while investigations incorporating analyses of multiple exposures have shown that associations observed with single exposures tend to be less pronounced [22]. A further constraint in the existing literature, which impedes the understanding of precise mechanisms, is the insufficient investigation into the mediating role of brain structure and function in the association between the environment and mental health.

To address these challenges, statistical models are needed that enable simultaneous modelling of high-dimensional data, aiming to reduce the complexity and understand underlying patterns by grouping them based on their shared characteristics and distinctions. These methods include independent component analysis, canonical correlation analysis, hierarchical clustering, latent class analyses, and normative modelling [236]. An example of such analyses has been demonstrated recently [110]. The authors analysed a comprehensive set of environmental variables such as pollution, area deprivation, greenspace and distance to various facilities, and reduced redundancy by applying confirmatory-factor analysis. Thereafter, sparce canonical correlation analysis was employed to identify complex living profiles related to distinct psychiatric symptom groups, while simultaneously allowing the qualitative and quantitative assessment of each factor and their contribution to risk or resilience. Finally, multiple sparse canonical correlation analysis explored the mediating role of brain morphology in the observed associations.

These findings lay the groundwork for understanding the biological processes involved in complex real-life environmental challenges. Further studies are needed to expand upon and provide deeper insights into the specific mechanisms and identify biomarkers for risk and resilience, using deeply phenotyped datasets. Moreover, the applicability of the findings should be examined across diverse populations, settings, and environmental conditions.

### Addressing long latency periods

Long latency periods may exist between exposure to environmental hazards and the onset of mental health or brain outcomes, making it further challenging to establish a clear cause–effect relationship. Most studies are cross-sectional and are based on a single MRI measurement. Repeated measurements across the lifespan could give insights into the temporal relationships and enable the examination of critical periods of vulnerability, windows of intervention, and long-term consequences of early-life exposure on later brain health. Therefore, longitudinal studies are crucial for examining these long-term trajectories of brain development, ageing and degeneration related to environmental exposures. Prominent examples of such studies include IMAGEN [237], ABCD (Adolescent Brain Cognitive Development) [238], Generation R [239], along with the ongoing follow-up assessments in the UK Biobank [240] and the NAKO (German National Cohort) [241]. Furthermore, longitudinal studies could help to assess pre- and post-exposure effects on brain outcomes. In this way, the immediate and delayed effects on the brain can be evaluated, as well as the potential reversibility or persistence of these effects.

### Enhancing macroenvironmental exposure assessment

Current literature relies on assessments of environmental factors that are based on a few stations or land use regression models which are spatially and temporally misaligned with the location or period of interest and may not capture accurately the level of environmental exposure. This issue is particularly important when studying susceptibility periods. Environmental exposures often vary in intensity, duration, and timing, posing additional challenges in their accurate measurement. Misclassification of environmental exposures might hinder small but clinically relevant associations or result in spurious associations. To improve the accuracy of exposure assessment, an increased granularity in the spatial and temporal resolution of data collection is required. Remote sensing satellite data, and integration of multiple data sources, such as air quality models and meteorological reanalysis data provide globally standardised environmental measures enabling the tracing of environmental features spanning back several decades [242–244]. The wealth of historical environmental data facilitates global comparative analyses and enables the assessment of the cumulative effects of environmental exposures. A recent study among young adults from China and Europe exemplified the application of several satellite-based measures of urbanicity to characterise spatiotemporal patterns of mental disorders risk [131]. Confirmatory factor analysis was performed to develop a composite urbanicity measure, which was calculated for each participant from birth to age of recruitment. This approach allowed to assess the cumulative effects and the susceptibility periods of lifetime urban exposure on brain and behaviours.

Measures of urbanicity or other features of macroenvironment that can be applied to different sociocultural conditions and geographies are significant, as they might uncover common associations with brain and behaviour and may assist in global public health policies and urban planning.

### Embracing mobility

Another source of misclassification is the static exposure assessment, disregarding that individuals are exposed to multiple environments along their daily movements. Considering the high spatial and temporal variability of some environmental exposures (e.g., pollutants associated with traffic and industrial production), the actual environmental exposure should be linked to the individual movement patterns and residence time to capture aetiological meaningful associations. Incorporating mobility patterns in data collection, such as daily movements, commuting behaviours, and residential relocations in combination with utilisation of geospatial techniques and geographic information systems will allow more accurate assessment of cumulative exposures. Furthermore, leveraging technology, such as wearable devices and mobile applications alongside ecological momentary assessments to collect real-time data on individuals’ environmental exposures may be helpful to overcome the ‘static assumption’ errors [245, 246]. By integrating sensors that measure parameters such as temperature, humidity, UV radiation, air pollution and activity levels, wearable devices provide a personalised perspective into the microclimates that individuals experience throughout their daily lives, accounting for factors such as indoor and outdoor environments and personal behaviours. This granular data allow the identification of patterns and correlations between atmospheric variables and their impact on mental well-being [247, 248].

### Consolidating future directions

To identify complex real-life environmental profiles and establish their relationship with brain and behaviour, a dataset with adequate overall power is essential. It can be achieved by increasing between-participant variations (combining study populations with heterogeneous macroenvironment and varying mental illness burden) and decreasing random measurement error (utilising objective measures of macroenvironment, repeated measurements and biomarkers). Driven by these objectives, a concerted effort is being made by the environMENTAL consortium, involving multidisciplinary expertise [236]. Through the integration of individual cross-sectional and longitudinal cohorts across Europe and beyond, the consortium aims to leverage the strength of existing datasets, which can be enriched with remote sensing, meteorological and air pollution data, and with digital-health tools enabling real-time data collection (i.e., smart phone applications and ecological momentary assessments). Furthermore, combining federated analyses, using the COINSTAC platform (Collaborative Informatics and Neuroimaging Suite Toolkit for Anonymous Computation) [249] with data harmonisation, and using representational biostatistical models, will enable the identification of impactful environmental signatures that can be evaluated for their replicability and generalisability across study designs, cultural settings, and molecular levels.

## Conclusions

The current review highlights that various macroenvironmental factors, including air pollution, neighbourhood disadvantage, and urbanicity, may alter brain structure and function and, consequently, mental health. Exposure to these factors, particularly during critical periods of development, might have lasting impacts, resulting in heightened risk for a range of mental illnesses. Then again, detrimental effects of urban environment related to higher risk for mental health disorders, like social stress and air pollution, might be attenuated with exposure to natural environments through decreased stress-related activation in brain regions for emotional regulation [144]. Similarly, higher safety norms may mitigate the harmful effects of regional socioeconomic adversity on brain and mental health [177].

However, our understanding of these interactions is still evolving and evidence on specific macroenvironmental factors, such as climate, noise and light pollution is sparse. The short-term and long-term effects of the macroenvironment on brain and mental health are elusive and the need for well-designed longitudinal analyses is pressing. The exploration of mediating and moderating factors, that explain these associations, not only in terms of brain but also, lifestyle and social factors, is essential. Additionally, there is a notable lack of studies on subpopulations and vulnerable groups.

By recognising the impact of environmental factors on brain plasticity processes, policymakers, and healthcare professionals can work towards creating healthier and more supportive environments that promote mental well-being and resilience.

## Supplementary information

Literature search and study selection
