# Supplementary material for: How does the macroenvironment influence brain and behaviour—a review of current status and future perspectives
Source: Mol Psychiatry. 2024 Apr 24;29(10):3268–86. doi: 10.1038/s41380-024-02557-x (PMC11449798; doi:10.1038/s41380-024-02557-x)
Supplement: Supplementary file 1 — Literature search and study selection [file 41380_2024_2557_MOESM1_ESM.pdf]

SUPPLEMENTARY INFORMATION

How does the macroenvironment influence brain and behaviour – a review of current status and future perspectives

Elli Polemiti, Ph.D.[1], Sören Hese, Ph.D.[2], Kerstin Schepanski, Ph.D.[3], Jiacan Yuan, Ph.D.[4], environMENTAL consortium*, Gunter Schumann, M.D.[1,5]

Affiliations:

[1] Centre of Population Neuroscience and Stratified Medicine (PONS), Department of Psychiatry and Clinical Neuroscience CCM, Charité-Universitätsmedizin Berlin, Germany

[2] Institute of Geography, Friedrich Schiller University Jena, Germany

[3] Institute of Meteorology, Free University Berlin, Germany

[4] Department of Atmospheric and Oceanic Sciences & Institute of Atmospheric Sciences & CMA-FDU Joint Laboratory of Marine Meteorology & IRDR-ICOE on Risk Interconnectivity and Governance on Weather/Climate Extremes Impact and Public Health, Fudan University, Shanghai, China

[5] Centre for Population Neuroscience and Stratified Medicine (PONS), Institute for Science and Technology of Brain-inspired Intelligence (ISTBI), Fudan University, Shanghai, China

* A list of authors and their affiliations appears at the end of the Supplementary Information

Search strategy and study selection

We conducted a literature search on the association between modalities of the macroenvironment and magnetic resonance imaging (MRI)-assessed brain structure and function in PubMed from January 1, 2010, to April 19, 2023. While different neuroimaging techniques offer unique advantages and insights, we focused on MRI studies due to our expertise in this area. We used pre-defined search terms (Supplementary Information), with no restrictions applied except for the filter [Humans]. In short, MeSH (Medical Subject Headings) terms and title/abstract text words related to environmental exposures were employed, including urbanisation, air, noise and light pollution, green space, blue space, regional socioeconomic factors, climate, weather extremes, combined with MRI-detected brain changes in structure and function. The reference lists of relevant systematic reviews identified in our formal search were hand-searched for relevant literature. Furthermore, studies known to the authors were added.

The present review is not exhaustive in covering all potential macroenvironmental factors influencing brain and mental health. Exclusion criteria were applied to focus on macroenvironmental factors that are pervasive and modifiable at the general population level. Publications on animal models and cell lines were excluded. Studies investigating the association between indoor air pollution, occupational hazards and neurotoxins with brain plasticity were further excluded due to their confinement in specific indoor environments, work-related settings, or lifestyle contexts. Finally, studies on natural disasters were excluded due to their sporadic occurrence and localised impact.

In the following tables (Supplemental Table 1 – 3) the full search strategy for PubMed is provided. All references obtained from the search in PubMed were imported in an excel file. An electronic search will first be undertaken with the excel file for identification of irrelevant records by using the terms: adenoma, angiopathy, atherosclero, bacteria, benign, breast, cancer, carcino, cardiac, cerebral palsy, cholera, Crohn, damage, dementia, dengue, diabetes, diarrhea, dysplasia, epilepsy, gut, hyperactivity, infarct, infect, infraction, injury, knee, lobectomy, lung, lymphoma, malaria, malignant, melanoma, metabolic, metastas, mice, microbio, mouse, myocarditis, necrosis, neoplasm, neurodegenerat, oncolog, pain, Parkinson, probiotic, prostate, pulmonary, rodent, sarcomas, sclerosis, stiffness, stimulation, stroke, thrombosis, tinnitus, tumor, tumour, vaccination.

Supplemental table 1. Search terms for studies relating to urban and natural environment, air pollution, climate, weather, and regional socioeconomic status.

#1	Environment	“Environment"[Mesh] OR "Geography"[Mesh] OR “Environmental Pollution"[Mesh] OR “physical environment*”[Title/Abstract] OR “living environment*”[Title/Abstract] OR “healthy environment*”[Title/Abstract] OR “unhealthy environment*”[Title/Abstract] OR “environmental factor*”[Title/Abstract] OR “environmental stressor*” [Title/Abstract] OR “environmental risk factor*”[Title/Abstract] OR “environmental adversity”[Title/Abstract] OR “environmental condition*” OR “environmental predictor*”[Title/Abstract] OR “environmental exposure*”[Title/Abstract] OR “environmental pollution”[Title/Abstract] OR “local environment*”[Title/Abstract] OR “environment pollution”[Title/Abstract]
#2	Urbanisation	Urbanization[Mesh] OR "Built Environment"[Mesh] OR "Environment Design"[Mesh] "Urban Population"[Mesh] OR "Suburban Population"[Mesh] OR "Rural Population"[Mesh] OR “built environment”[Title/Abstract] OR "Environment Design"[Title/Abstract] OR urban*[Title/Abstract] OR city[Title/Abstract] OR cities[Title/Abstract] town*[Title/Abstract] OR rural*[Title/Abstract] OR suburb*[Title/Abstract] OR downtown[Title/Abstract] OR “non-urban”[Title/Abstract] OR “remote communit*”[Title/Abstract] OR “remote area*”[Title/Abstract] OR “remote region*”[Title/Abstract] OR “isolated communit*”[Title/Abstract] OR “isolated area*”[Title/Abstract] OR “isolated region*”[Title/Abstract] OR “gray space*”[Title/Abstract] OR “grey space*”[Title/Abstract] OR “impervious surface*”[Title/Abstract] OR “impervious area*”[Title/Abstract] OR “impervious surface”[Title/Abstract:~2]
#3	Light Pollution	"Light Pollution"[MeSH] OR "Lighting"[Mesh] OR “light pollution”[Title/Abstract] OR “light at night*”[Title/Abstract] OR LAN[Title/Abstract] OR “artificial light*”[Title/Abstract] OR “night light*”[Title/Abstract] OR “light exposure*” [Title/Abstract] OR “outdoor light*”[Title/Abstract] OR “outdoor light”[Title/Abstract:~2]
#4	Noise	("Noise"[Mesh] OR “noise pollution”[Title/Abstract] OR “acoustic pollution”[Title/Abstract] OR “aeroplane noise”[Title/Abstract:~2] OR “environmental noise*”[Title/Abstract] OR “environment noise*”[Title/Abstract] OR “aircraft noise*”[Title/Abstract] OR “noise* exposure”[Title/Abstract] OR “ambient noise*” [Title/Abstract] OR “transport noise”[Title/Abstract:~2] OR “transportation noise”[Title/Abstract:~2] OR “residential noise”[Title/Abstract:~2] OR “industrial noise”[Title/Abstract:~2] OR “neighborhood noise”[Title/Abstract:~2] OR “neighbourhood noise”[Title/Abstract:~2] OR “noise level*”[Title/Abstract] OR “urban noise”[Title/Abstract:~2] OR “jet noise”[Title/Abstract:~2] OR “noise disturbance*”[Title/Abstract] OR “community noise”[Title/Abstract:~2] OR “vehicle noise”[Title/Abstract:~2] OR “noise metric*”[Title/Abstract] OR “noise exposure*”[Title/Abstract] OR “noise ind*”[Title/Abstract] OR “turbine noise”[Title/Abstract:~2] OR “freeway noise”[Title/Abstract:~2] OR “highway noise”[Title/Abstract:~2] OR “industry noise”[Title/Abstract:~2] OR “road noise”[Title/Abstract:~2] OR “motorway noise”[Title/Abstract:~2]) NOT “occupational”[Title/Abstract]
#5	Green space	"Nature"[Mesh] OR "Forests"[Mesh] OR "Parks, Recreational"[Mesh] OR “natural environment*”[Title/Abstract] OR “nature exposure*”[Title/Abstract] OR “environmental nature”[Title/Abstract:~10] OR “nature-based”[Title/Abstract] OR “natural land*”[Title/Abstract] OR “natural space*”[Title/Abstract] OR “natural area*”[Title/Abstract] OR “natural setting*”[Title/Abstract] OR “nature reserve”[Title/Abstract] OR “green space*”[Title/Abstract] OR

		greenspace*[Title/Abstract] OR “green area*”[Title/Abstract] OR greenness[Title/Abstract] OR greenery[Title/Abstract] OR vegetation*[Title/Abstract] OR "tree cover"[Title/Abstract:~2] OR "tree canopy"[Title/Abstract:~2] OR "tree density"[Title/Abstract:~2] OR "tree volume"[Title/Abstract:~4] OR "tree area*"[Title/Abstract:] OR "leaf area*"[Title/Abstract] OR "plant area*"[Title/Abstract] OR "park"[Title/Abstract] OR "parks"[Title/Abstract] OR ((forest[Title/Abstract] OR forests[Title/Abstract] OR forest-based[Title/Abstract] OR “forested area*”[Title/Abstract]) NOT (“forest plot*”[Title/Abstract] OR “random forest*”[Title/Abstract])) OR outdoor*[Title/Abstract] OR “open space*”[Title/Abstract] OR “wilderness”[Title/Abstract] OR “wild land”[Title/Abstract] OR “wild space*”[Title/Abstract] OR “woodland*”[Title/Abstract] OR “land use*”[Title/Abstract] OR “land cover”[Title/Abstract]
#6	Blue space	"Water"[Mesh] OR "Rivers"[Mesh] OR "Oceans and Seas"[Mesh] OR "Wetlands"[Mesh] OR “blue space*”[Title/Abstract] OR “bluespace*”[Title/Abstract] OR “blue area*”[Title/Abstract] OR “blue region*”[Title/Abstract] OR “coast*”[Title/Abstract] OR “wetland*”[Title/Abstract] “freshwater*”[Title/Abstract] OR “lake*”[Title/Abstract] OR “blue bodies”[Title/Abstract] OR “water space*”[Title/Abstract] OR “water bodies”[Title/Abstract] OR “water area*”[Title/Abstract] OR “water region*”[Title/Abstract] OR “water feature*”[Title/Abstract] OR “waterway*”[Title/Abstract] OR “water environment*”[Title/Abstract] OR “sea”[Title/Abstract] OR “river”[Title/Abstract] OR “rivers”[Title/Abstract] OR “riverbank*”[Title/Abstract] OR “riverside*”[Title/Abstract] OR “marine area*”[Title/Abstract] OR “marine environment*”[Title/Abstract] OR “marine region*”[Title/Abstract] OR “aquatic area*”[Title/Abstract] OR “aquatic environment*”[Title/Abstract] OR “aquatic region*”[Title/Abstract] OR “waterscapes”[Title/Abstract] OR “inland water”[Title/Abstract:~2] OR “beach*”[Title/Abstract] OR “pond”[Title/Abstract] OR “ponds” [Title/Abstract]
#7	Regional Socioeconomic status	"Residence Characteristics"[Mesh] OR "Housing/standards"[MAJR] OR "Housing Quality"[Mesh] OR "Social Environment"[Mesh] OR "Neighborhood Characteristics"[Mesh] OR "Poverty Areas"[Mesh] OR "residence characteristic*"[Title/Abstract] OR "residential characteristic*"[Title/Abstract] OR “community characteristic*”[Title/Abstract] OR “community deprivation”[Title/Abstract:~3] OR "residential deprivation"[Title/Abstract:~3] OR "residence deprivation"[Title/Abstract:~3] OR "deprivation index"[Title/Abstract] OR “community deprivation”[Title/Abstract:~2] OR neighborhood*[Title/Abstract] OR neighbourhood*[Title/Abstract] OR “residence area*”[Title/Abstract] OR “residential area*”[Title/Abstract] OR “residential environment”[Title/Abstract] OR “residential deprivation”[Title/Abstract:~2] OR “residence deprivation”[Title/Abstract:~2] OR “area deprivation”[Title/Abstract:~2] OR “deprivation areas”[Title/Abstract:~2] OR “deprivation area”[Title/Abstract:~2] OR ghetto*[Title/Abstract] OR slum*[Title/Abstract] OR “poverty area*”[Title/Abstract] OR “neighborhood poverty”[Title/Abstract:~2] OR “neighbourhood poverty”[Title/Abstract:~2] OR “community poverty”[Title/Abstract:~2] OR “poverty region*”[Title/Abstract] OR “regional poverty”[Title/Abstract:~2] OR “local poverty”[Title/Abstract:~2] OR “poor area*”[Title/Abstract] OR “poor region*”[Title/Abstract] OR “low income area*”[Title/Abstract] OR

		“low income region*”[Title/Abstract] OR “middle income area*”[Title/Abstract] OR “middle income region*”[Title/Abstract] OR “high income area*”[Title/Abstract] OR “high income region*”[Title/Abstract] OR “social inequ*”[Title/Abstract] OR “economic inequ*”[Title/Abstract] OR “social equit*”[Title/Abstract] OR “economic equit*”[Title/Abstract] OR “social equal*”[Title/Abstract] OR “economic equal*”[Title/Abstract] OR “developing area*”[Title/Abstract] OR “developing region*”[Title/Abstract] OR “local crim*”[Title/Abstract] OR “local crime”[Title/Abstract:~2] OR “area crime”[Title/Abstract:~2] OR “area crim*”[Title/Abstract] OR “community crime”[Title/Abstract:~2] OR “community crim*”[Title/Abstract] OR “crime hot spot*”[Title/Abstract] OR “local safety”[Title/Abstract] OR “area safety”[Title/Abstract:~2] OR “community safety”[Title/Abstract:~2] OR “local violence”[Title/Abstract:~2] OR “area violence”[Title/Abstract:~2] OR “community violence”[Title/Abstract:~2] OR “local socioeconomic”[Title/Abstract:~2] OR “area socioeconomic”[Title/Abstract:~2] OR “community socioeconomic”[Title/Abstract:~2]
#8	Land use/ cover	“Land use”[Title/Abstract] OR “land cover”[Title/Abstract] OR walkability[Title/Abstract] OR walkable[Title/Abstract]
#9	Population density	"Population Density"[Mesh] OR “population densit*”[Title/Abstract] OR overpopulation[Title/Abstract] OR “population size”[Title/Abstract]
#10	Climate/ weather	Climate[Mesh] OR "Climate Change"[Mesh] OR "Extreme Heat"[Mesh] OR "Weather"[Mesh] OR "Extreme Cold Weather"[Mesh] OR "Extreme Hot Weather"[Mesh] OR "Extreme Weather"[Mesh] OR climat*[Title/Abstract] OR weather[Title/Abstract] OR “Extreme Heat”[Title/Abstract] OR "Ambient Temperature*"[Title/Abstract] OR "Air Temperature*"[Title/Abstract] OR "Hot Temperature*"[Title/Abstract] OR "Cold Temperature*"[Title/Abstract] OR “Heat extreme*”[Title/Abstract] OR "Extreme Environment*"[Title/Abstract] OR meteorolog*[Title/Abstract] OR “warming”[Title/Abstract] OR “environmental crisis”[Title/Abstract] OR “climate crisis”[Title/Abstract] OR “greenhouse”[Title/Abstract] OR “heatwave*”[Title/Abstract] OR “heat wave*”[Title/Abstract] OR “cold wave*”[Title/Abstract]
#11	Rainfall	"Humidity"[Mesh] OR "Rain"[Mesh] OR "Snow"[Mesh] OR “rainfall*”[Title/Abstract] OR “rain”[Title/Abstract] OR “humidity”[Title/Abstract] OR “snow”[Title/Abstract] OR “precipitation*”[Title/Abstract] OR “wet day*”[Title/Abstract] OR "Floods"[Mesh] OR “flood*”[Title/Abstract] OR “storm*”[Title/Abstract]
#12	Air pollution	"Air Pollutants"[Mesh] OR "Particulate Matter"[Mesh] OR "Air Pollution"[Mesh] OR "Gases"[Mesh] OR "Volatile Organic Compounds"[Mesh] OR "Sulfur Oxides"[Mesh] OR "Ozone"[Mesh] OR “atmospheric composition”[Title/Abstract] OR “atmosphere composition”[Title/Abstract:~2] OR “air pollutant*”[Title/Abstract] OR “air pollution”[Title/Abstract] OR “particulate matter”[Title/Abstract] OR “PM2.5”[Title/Abstract] OR “PM10”[Title/Abstract] OR “PM(2.5)”[Title/Abstract] OR “PM(10)”[Title/Abstract] OR “ammonia”[Title/Abstract] OR “carbon oxide*”[Title/Abstract] OR “CO2”[Title/Abstract] “CO(2)”[Title/Abstract] OR “carbon monoxide”[Title/Abstract] OR “carbon dioxide”[Title/Abstract] OR “nitrogen oxide*”[Title/Abstract] OR “nitrogen dioxide”[Title/Abstract] OR “NOx”[Title/Abstract] OR “NO2”[Title/Abstract] OR “NO(x)”[Title/Abstract] OR “NO(2)”[Title/Abstract] OR

		"chlorine"[Title/Abstract] OR "sulfur dioxide"[Title/Abstract] OR "sulphur dioxide"[Title/Abstract] OR "SO2"[Title/Abstract] OR "SO(2)"[Title/Abstract] OR "ozone"[Title/Abstract] OR "O3"[Title/Abstract] OR "volatile organic compound*"[Title/Abstract] OR "VOC"[Title/Abstract] OR "black carbon"[Title/Abstract] OR "polycyclic aromatic hydrocarbons"[Title/Abstract] OR "Aerosol optical depth"[Title/Abstract] OR "AOD"[Title/Abstract] OR "formaldehyde"[Title/Abstract] OR "dust aerosols"[Title/Abstract:~2] OR "particle*"[Title/Abstract] OR "traffic*"[Title/Abstract] OR "ambient*"[Title/Abstract] OR "emission*"[Title/Abstract] OR "outdoor air quality"[Title/Abstract] OR "exhaust gases"[Title/Abstract:~2] OR "vehicle exhaust"[Title/Abstract:~2] OR "exhaust fumes"[Title/Abstract] OR "vehicle fumes"[Title/Abstract:~2] OR "freeway fumes"[Title/Abstract:~2] OR "highway fumes"[Title/Abstract:~2] OR "motorway fumes"[Title/Abstract:~2] OR "road fumes"[Title/Abstract:~2] OR "vehicle gases"[Title/Abstract:~2] OR "freeway gases"[Title/Abstract:~2] OR "highway gases"[Title/Abstract:~2] OR "motorway gases"[Title/Abstract:~2] OR "road gases"[Title/Abstract:~2]
#13	Sunlight/ cloud cover	"Sunlight"[Mesh] OR "sunlight"[Title/Abstract] OR "daylight"[Title/Abstract] OR "sun time"[Title/Abstract:~2] OR "sunshine"[Title/Abstract] OR cloud*[Title/Abstract] OR "sun radiation"[Title/Abstract]
#14	Combine	#1 OR #2 OR #3 OR #4 OR #5 OR #6 OR #7 OR #8 OR #9 OR #10 OR #11 OR #12 OR #13
#15	Exclusion criteria	("Animals"[Mesh] OR "Gastrointestinal Microbiome"[Mesh] OR "Rats"[Mesh] OR COVID-19[Mesh] OR "SARS-CoV-2"[Mesh]) NOT Humans[Mesh]
#16	Combine	#14 NOT #15

Supplemental table 2. Search terms for studies relating to MRI-detected changes in brain structure and function.

#17	Brain	(("Brain"[Mesh] OR "Neurosciences"[Mesh] OR "brain"[Title/Abstract] OR "limbic system"[Title/Abstract] OR "neuroscience"[Title/Abstract]) NOT ("brain cancer*"[Title/Abstract] OR "brain tumor*"[Title/Abstract] OR "brain tumour*"[Title/Abstract] OR "brain injur*"[Title/Abstract] OR "Neurodegenerative Diseases"[Mesh] OR "Heredodegenerative Disorders, Nervous System"[Mesh]))
#18	Neuroimaging	"Neuroimaging"[Mesh] OR "Magnetic Resonance Imaging"[Mesh] OR "neuroimag*"[Title/Abstract] OR "brain imag*"[Title/Abstract] OR "diffusion tensor"[Title/Abstract] OR "diffusion tractography"[Title/Abstract] OR "DTI"[Title/Abstract] OR "magnetic resonance"[Title/Abstract] OR "MRI"[Title/Abstract] OR "fMRI*"[Title/Abstract] OR "functional magnetic"[Title/Abstract] OR "sMRI*"[Title/Abstract] OR "structural magnetic"[Title/Abstract] OR "rs-fMRI*"[Title/Abstract] OR "resting state*"[Title/Abstract] OR "FLAIR"[Title/Abstract] OR "3dFLAIR"[Title/Abstract] OR "fluid attenuated inversion recovery"[Title/Abstract] OR "T1-w"[Title/Abstract] OR "T2-w"[Title/Abstract] OR "T1-weight*"[Title/Abstract] OR "T2-weight*"[Title/Abstract] OR "T1W"[Title/Abstract] OR "T2W"[Title/Abstract] OR "weighted imag*"[Title/Abstract] OR "Freesurfer"[Title/Abstract]

#19	Brain areas	“limbic thalam*”[Title/Abstract] OR “Hippocamp*”[Title/Abstract] OR “parahippocamp*”[Title/Abstract] OR “subiculum*”[Title/Abstract] OR ((“CA1”[Title/Abstract] OR “CA-1”[Title/Abstract] OR “CA2”[Title/Abstract] OR “CA-2”[Title/Abstract] OR “CA3”[Title/Abstract] OR “CA-3”[Title/Abstract]) AND (“region*”[Title/Abstract] OR “area*”[Title/Abstract])) OR ((“dentate”[Title/Abstract]) AND (“gyrus”[Title/Abstract] OR “region*”[Title/Abstract] OR “area*”[Title/Abstract] OR “fasc*”[Title/Abstract])) OR “hypothalam*”[Title/Abstract] OR “limbic lobe*”[Title/Abstract] OR “gyrus cinguli”[Title/Abstract] OR “perforant pathway”[Title/Abstract] OR “septum brain”[Title/Abstract:~2] OR “septal nuclei”[Title/Abstract] OR “septum pellucidum”[Title/Abstract] OR “amygdala”[Title/Abstract] OR “basolateral nuclear complex”[Title/Abstract] OR “central amygdaloid nucleus”[Title/Abstract] OR “corticomedial nuclear complex”[Title/Abstract] OR “epithalamus”[Title/Abstract] OR “habenula”[Title/Abstract] OR “pineal gland”[Title/Abstract] OR “insular cortex”[Title/Abstract] OR ((“orbito*”[Title/Abstract] OR “front*”[Title/Abstract] OR “pariet*”[Title/Abstract] OR “tempo*”[Title/Abstract] OR “occipit*”[Title/Abstract] OR “arterio*”[Title/Abstract]) AND (“lobe”[Title/Abstract] OR “region*”[Title/Abstract] OR “area*”[Title/Abstract] OR “cortex”[Title/Abstract] OR “ROI”[Title/Abstract] OR “tissue*”[Title/Abstract])) OR “grey matter”[Title/Abstract] OR “gray matter”[Title/Abstract] OR “white matter”[Title/Abstract] OR “subcortex”[Title/Abstract] OR “sub-cortex”[Title/Abstract] OR “cerebell*”[Title/Abstract] OR “cortical”[Title/Abstract] OR “subcortical”[Title/Abstract] OR “sub-cortical”[Title/Abstract] OR “cerebral”[Title/Abstract] OR “cingulate”[Title/Abstract] OR “gyrus cinguli”[Title/Abstract] OR “neocort*”[Title/Abstract] OR “postrhinal*”[Title/Abstract] OR “perirhinal*”[Title/Abstract] OR “uncinate fasciculus”[Title/Abstract]
#20	Combine	#17 OR #18 OR #19

Supplemental table 3. Search terms for studies relating to the macroenvironment and MRI-detected changes in brain structure and function.

#21	Environment and brain	#16 AND #20
#22	Apply filter	#21 AND Humans[Filter]

environMENTAL consortium

Elli Polemiti[1], Esther Hitchen[1], Hedi Kebir[1], Tristram Lett[1], Nilakshi Vaidya[1], Jean-Charles Roy[1], Sören Hese[2], Paul Renner[2], Kerstin Schepanski[3], Jiacan Yuan[4], Gunter Schumann[1,5], Tianye Jia[5], Xiao Chang[5], Yuxiang Dai[5], Yunman Xia[5], Henrik Walter[6], Andreas Heinz[6,7], Emin Serin[6], Markus Ralser[8], Sven Twardziok[9], Roland Eils[9], Marcel Jentsch[9], Ulrike Helene Taron[9], Tatjana Schütz[9], Tobias Banaschewski[10], Maja Neidhart[6,10], Nathalie E. Holz[10], Nina Christmann[10], Karina Jansone[10], Andreas Meyer-Lindenberg[11], Heike Tost[11], Emanuel Schwarz[11,12], Frauke Nees[13], Sebastian Siehl[13], Stephan Lehmler[13], Ole A. Andreassen[14], Lars T. Westlye[14,15], Dennis van der Meer[14,16], Sara Fernández-Cabello[14,15], Rikka Kjelkenes[14], Michael Rapp[17], Mira Tschorn[17], Sarah J. Böttger[17], Andre Marquand[18], Antoine Bernas[18], Gaia Novarino[19], Mel Slater[20,21], Jaime Gallego[20], Álvaro Pastor[20], Peter Sommer[22], Karen Schmitt[22], Johannes H. Wilbertz[22], Viktor Jirsa[23], Spase Petkoski[23], Anastasios-Polykarpos Athanasiadis[23], Vince D. Calhoun[24], Nicholas Clinton[25], Sylvane Desrivières[26], Kofoworola Agunbiade[26], Xinyang Yu[26], Zuo Zhang[26], Di Chen[26], Georgie Keggin[26], Ameli Schwalber[27], Dennis Cleff[27], Bernd Carsten Stahl[28], George Ogoh[28], Tamara Schikowski[29], Ragnhild Eek Brandlistuen[30], Guillem Feixas[31,32], Francisco J. Eiroa-Orosa[31,32], Per Hoffmann[33], Markus M. Nöthen[33], Andreas J. Forstner[33], Abigail Miller[33], Carina M. Mathey[33], Isabelle Claus[33], Stefanie Heilmann-Heimbach[33], Yuzhu Li[34], Yanqing Zhang[35]

Affiliations:

[1]Centre of Population Neuroscience and Stratified Medicine (PONS), Department of Psychiatry and Clinical Neuroscience CCM, Charité-Universitätsmedizin Berlin, Berlin, Germany.

[2]Institute of Geography, Friedrich Schiller University Jena, Jena, Germany.

[3]Institute of Meteorology, Free University Berlin, Berlin, Germany.

[4]Department of Atmospheric and Oceanic Sciences & Institute of Atmospheric Sciences & CMA-FDU Joint Laboratory of Marine Meteorology & IRDR-ICOE on Risk Interconnectivity and Governance on Weather/Climate Extremes Impact and Public Health, Fudan University, Shanghai, China.

[5]Centre for Population Neuroscience and Stratified Medicine (PONS), Institute for Science and Technology of Brain-inspired Intelligence (ISTBI), Fudan University, Shanghai, China.

[6]Department of Psychiatry and Psychotherapy CCM, Charité-Universitätsmedizin Berlin, Berlin, Germany.

[7]German Center for Mental Health (DZPG), Partner Site Berlin-Potsdam, Germany

[8]Institute of Biochemistry Charité-Universitätsmedizin Berlin, Berlin, Germany.

[9]Berlin Institute of Health at Charité-Universitätsmedizin Berlin, Berlin, Germany.

[10]Department of Child and Adolescent Psychiatry and Psychotherapy, Central Institute of Mental Health, Medical Faculty Mannheim/Heidelberg University, Mannheim, Germany.

[11]Department of Psychiatry and Psychotherapy, Central Institute of Mental Health, Medical Faculty Mannheim/Heidelberg University, Mannheim, Germany.

[12]Hector Institute for Artificial Intelligence in Psychiatry, Central Institute of Mental Health, Medical Faculty Mannheim/Heidelberg University, Mannheim, Germany.

[13]Institute of Medical Psychology and Medical Sociology, University Medical Center Schleswig Holstein, Kiel University, Kiel, Germany.

[14]Centre for Precision Psychiatry, Division of Mental Health and Addiction, Oslo University Hospital & Institute of Clinical Medicine, University of Oslo, Oslo, Norway.

[15]Department of Psychology, University of Oslo, Oslo, Norway.

[16]School of Mental Health and Neuroscience, Faculty of Health, Medicine and Life Sciences, Maastricht University, The Netherlands.

[17]Department for Social and Preventive Medicine, University of Potsdam, Potsdam, Germany.

[18]Donders Institute/Radboud UMC, Nijmegen, the Netherlands.

[19]Institute of Science and Technology Austria (ISTA), Klosterneuburg, Austria.

[20]Campus de Mundet, ICREA-University of Barcelona, Barcelona, Spain.

[21]Department of Computer Science, University College London, London, United Kingdom.

[22]Ksilink, Strasbourg, France.

[23]Institut National de la Santé et de la Recherche Médicale (Inserm), Institut de Neurosciences des Systèmes (INS) UMR1106, Aix Marseille Université, Marseille, France.

[24]Tri-institutional Center for Translational Research in Neuroimaging and Data Science (TReNDS), Georgia State, Georgia Tech, Emory, Atlanta, Georgia.

[25]Google, Inc, Mountain View, California.

[26]Institute of Psychiatry, Psychology & Neuroscience, SGDP Centre, King's College London, London, United Kingdom.

[27]Concentris research management GmbH.

[28]School of Computer Science, University of Nottingham, Nottingham, United Kingdom.

[29]IUF – Leibniz Research Institute for Environmental Medicine, Düsseldorf, Germany.

[30]Norwegian Institute of Public Health, Oslo, Norway.

[31]Department of Clinical Psychology and Psychobiology, Universitat de Barcelona, Barcelona, Spain.

[32]Institute of Neurosciences, Universitat de Barcelona, Barcelona, Spain.

[33]Institute of Human Genetics, University Hospital of Bonn, Bonn, Germany.

[34]Institute of Science and Technology for Brain-Inspired Intelligence, Fudan University, Shanghai, China.

[35]Institutes of Biomedical Sciences, Fudan University, Shanghai, China.

REFERENCES

1. Clifford A, Lang L, Chen R, Anstey KJ, Seaton A. Exposure to air pollution and cognitive functioning across the life course - A systematic literature review. Environ Res. 2016;147:383–98.

2. Hong C, Efferth T. Systematic Review on Post-Traumatic Stress Disorder Among Survivors of the Wenchuan Earthquake. Trauma Violence Abuse. 2016;17(5):542–61.

3. Power MC, Adar SD, Yanosky JD, Weuve J. Exposure to air pollution as a potential contributor to cognitive function, cognitive decline, brain imaging, and dementia: A systematic review of epidemiologic research. Neurotoxicology. 2016;56:235–53.

4. Mothersill O, Donohoe G. Neural effects of social environmental stress – an activation likelihood estimation meta-analysis. Psychol Med. 2016 Jul 24;46(10):2015–23.

5. Misiak B, Stramecki F, Gawęda Ł, Prochwicz K, Sąsiadek MM, Moustafa AA, et al. Interactions Between Variation in Candidate Genes and Environmental Factors in the Etiology of Schizophrenia and Bipolar Disorder: a Systematic Review. Mol Neurobiol. 2018;55(6):5075–100.

6. Buckley L, Broadley M, Cascio CN. Socio-economic status and the developing brain in adolescence: A systematic review. Child Neuropsychol. 2019 Oct 3;25(7):859–84.

7. Shuda Q, Bougoulias ME, Kass R. Effect of nature exposure on perceived and physiologic stress: A systematic review. Complement Ther Med. 2020;53:102514.

8. Lopuszanska U, Samardakiewicz M. The Relationship Between Air Pollution and Cognitive Functions in Children and Adolescents: A Systematic Review. Cogn Behav Neurol. 2020;33(3):157–78.

9. Chandra M, Rai CB, Kumari N, Sandhu VK, Chandra K, Krishna M, et al. Air Pollution and Cognitive Impairment across the Life Course in Humans: A Systematic Review with Specific Focus on Income Level of Study Area. Int J Environ Res Public Health. 2022;19(3):1405.

10. Bolouki A. Neurobiological effects of urban built and natural environment on mental health: systematic review. Rev Environ Health. 2023;38(1):169–79.

11. Balboni E, Filippini T, Crous-Bou M, Guxens M, Erickson LD, Vinceti M. The association between air pollutants and hippocampal volume from magnetic resonance imaging: A systematic review and meta-analysis. Environ Res. 2022;204(Pt A):111976.

12. Sprague NL, Bancalari P, Karim W, Siddiq S. Growing up green: a systematic review of the influence of greenspace on youth development and health outcomes. J Expo Sci Environ Epidemiol. 2022;32(5):660–81.

13. Zundel CG, Ryan P, Brokamp C, Heeter A, Huang Y, Strawn JR, et al. Air pollution, depressive and anxiety disorders, and brain effects: A systematic review. Neurotoxicology. 2022;93:272–300.

14. Fowler CH, Bagdasarov A, Camacho NL, Reuben A, Gaffrey MS. Toxicant exposure and the developing brain: A systematic review of the structural and functional MRI literature. Neurosci Biobehav Rev. 2023;144:105006.

15. de Prado Bert P, Mercader EMH, Pujol J, Sunyer J, Mortamais M. The Effects of Air Pollution on the Brain: a Review of Studies Interfacing Environmental Epidemiology and Neuroimaging. Curr Environ Health Rep. 2018;5(3):351–64.
